# Supplementary material for: Host-encoded DNA methyltransferases modify the epigenome and host tropism of invading phages
Source: iScience. 2025 Mar 22;28(4):112264. doi: 10.1016/j.isci.2025.112264 (PMC12003011; doi:10.1016/j.isci.2025.112264)
Supplement: Document S1. Figures S1–S5 [file mmc1.pdf]

iScience, Volume *28*

Supplemental information

Host-encoded DNA methyltransferases modify the epigenome and host tropism of invading phages

Michiko Takahashi, Satoshi Hiraoka, Yuki Matsumoto, Rikako Shibagaki, Takako Ujihara, Hiromichi Maeda, Satoru Seo, Keizo Nagasaki, Hiroaki Takeuchi, and Shigenobu Matsuzaki

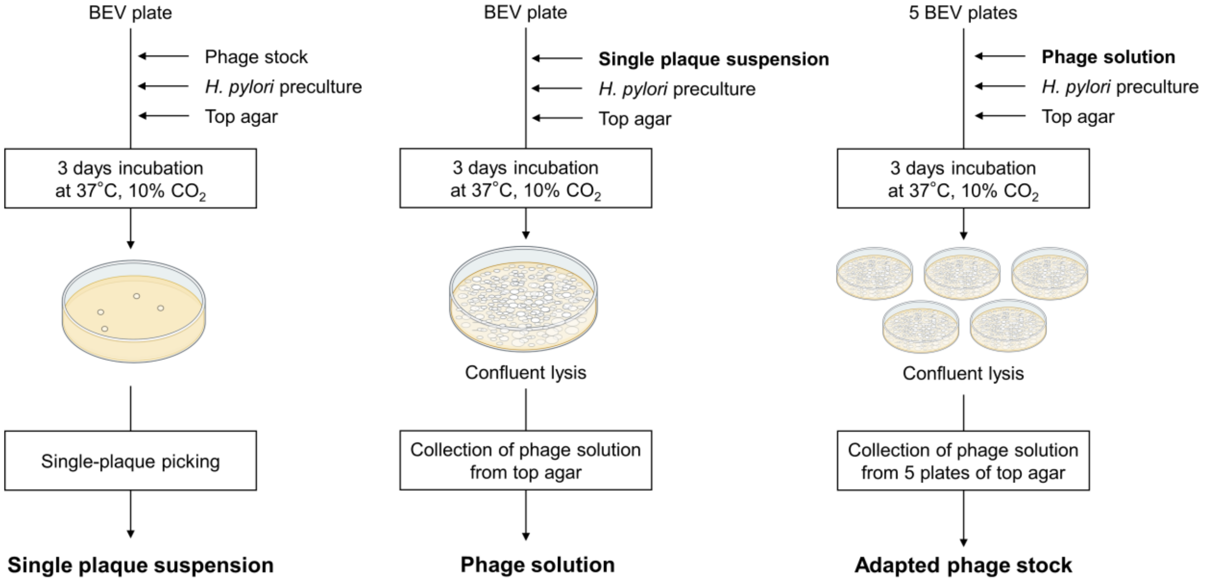

Figure S1. Overview of preparation procedure for adapted phages.

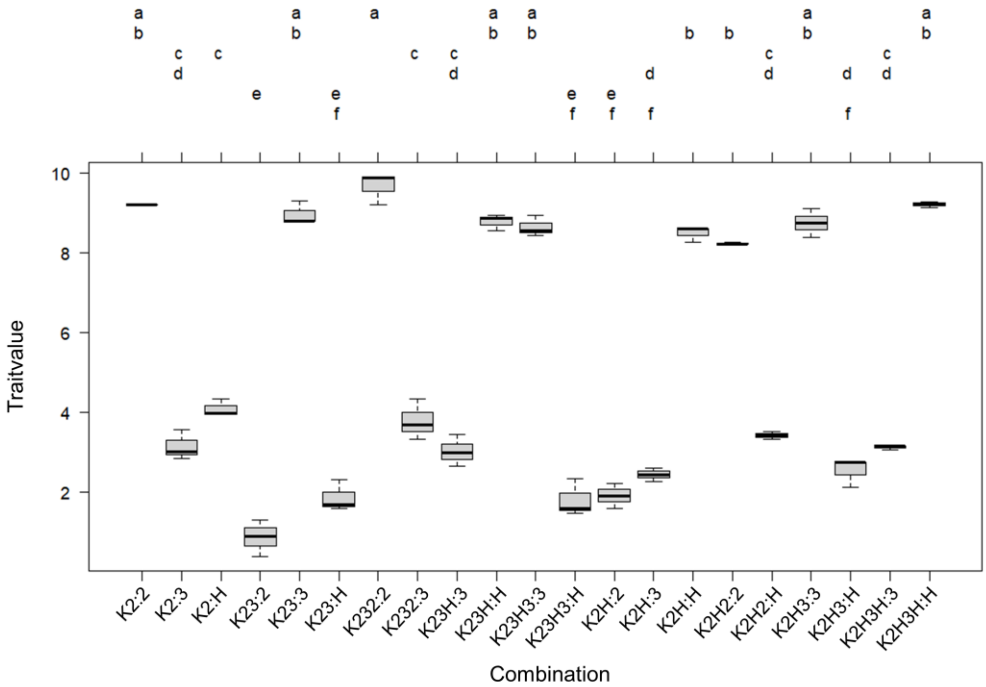

Figure S2. Tukey-Kramer pairwise comparisons of phage titers. Corresponding titers are shown in Figure 2. Combination of phage and host strain are indicated as 'adapted phage': 'host strain used for titration' format in the x-axis: for example, 'K2:2' means a titer of K2 against *H. pylori* 26695. Solid black bar within each box represents median. Upper alphabet summarizes the results of the Tukey-Kramer test; letters denote significant differences ($p < 0.01$) in mean titers with groups a > b > c > d > e > f.

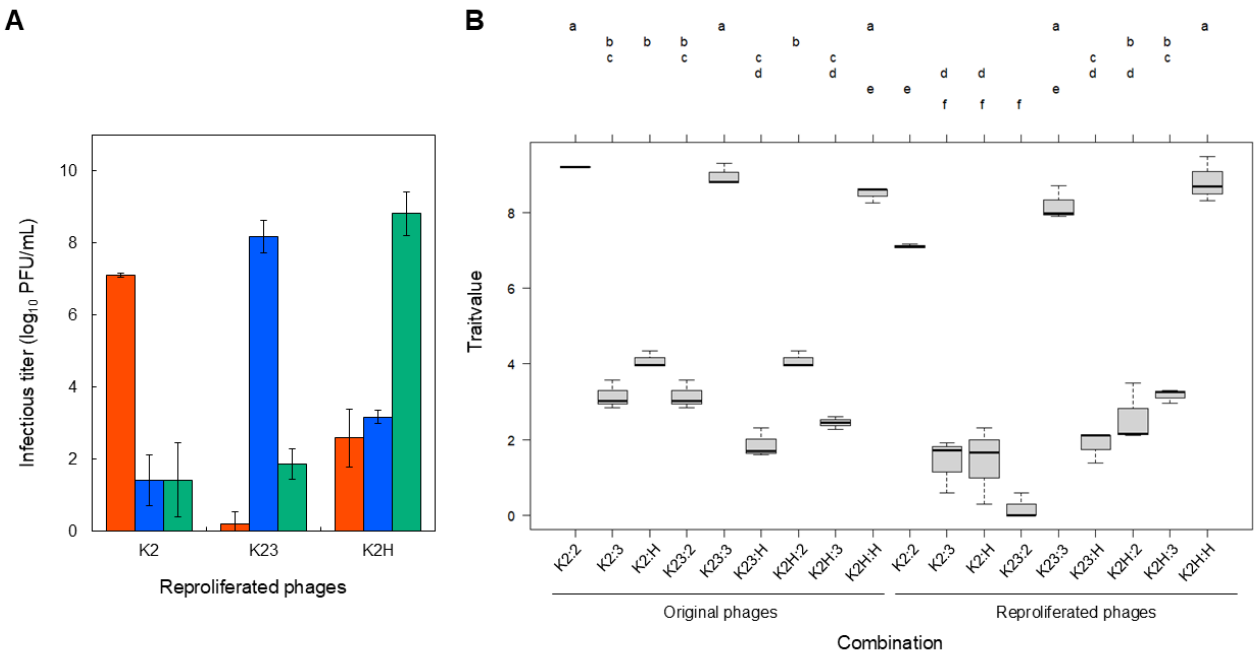

Figure S3. Infectious titer of reproliferated phages. (A) Orange, blue, and green bars represent titer of reproliferated KHP30T phages to *H. pylori* 26695, 3401T, and HPK5, respectively. Error bars indicate standard deviation (n = 3). (B) Tukey-Kramer pairwise comparisons of reproliferated phage titers. Phage and host combinations are indicated in the same format as Figure S2. Infectious titer of the 'Original phages' is derived from Figure 2. Solid black bar within each box represents median. Upper alphabet summarizes the results of the Tukey-Kramer test; letters denote significant differences (p < 0.01) in mean titers with groups a > b > c > d > e > f.

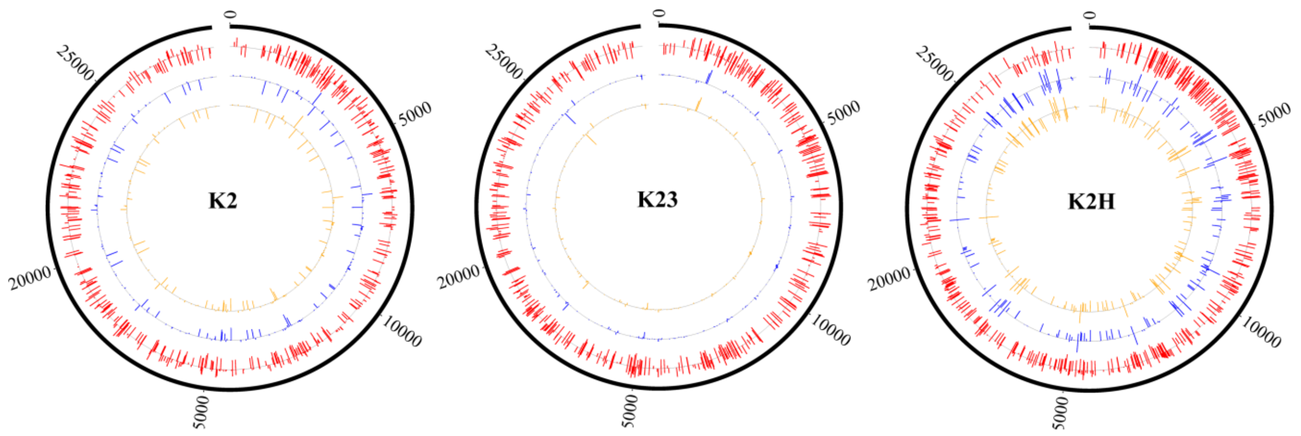

Figure S4. Methylomes of adapted phages. Circos plot showing the distribution of DNA methylation. Orange, blue, and red bars represent m4C, m5C, and m6A modifications, respectively. K2 and K23 were sequenced using PacBio Sequel II in CCS mode, whereas K2H was sequenced using PacBio Sequel in CLR mode.

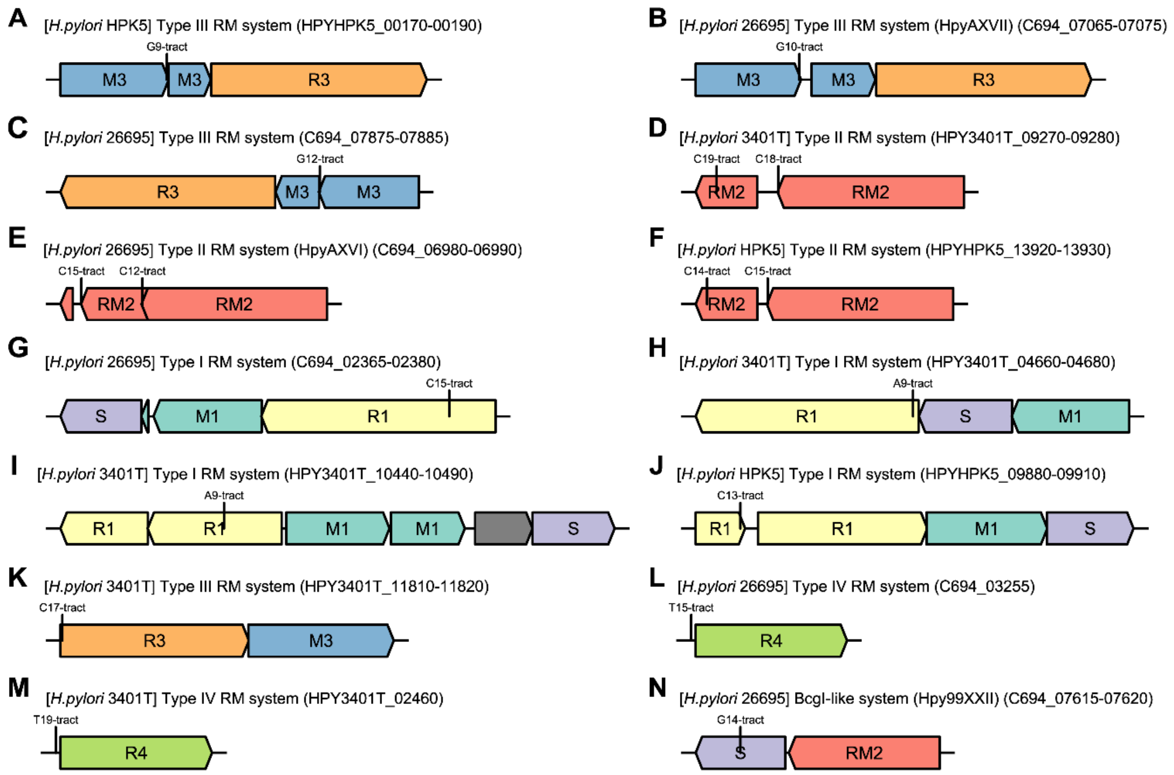

Figure S5. Genetic organization of RM system genes associated with SSRs. Homopolymeric tracts inside or upstream of RM genes were specified as SSRs. RM gene types are indicated by color and text inside to each CDS. CDSs not assigned to RM system genes are colored in gray.
